# Characteristics and outcomes of frail patients with suspected infection in intensive care units: a descriptive analysis from a multicenter cohort study

**DOI:** 10.1186/s12877-020-01893-1

**Published:** 2020-11-20

**Authors:** Akira Komori, Toshikazu Abe, Kazuma Yamakawa, Hiroshi Ogura, Shigeki Kushimoto, Daizoh Saitoh, Seitaro Fujishima, Yasuhiro Otomo, Joji Kotani, Yuichiro Sakamoto, Junichi Sasaki, Yasukazu Shiino, Naoshi Takeyama, Takehiko Tarui, Ryosuke Tsuruta, Taka-aki Nakada, Toru Hifumi, Hiroki Iriyama, Toshio Naito, Satoshi Gando, for the JAAM SPICE Study Group

**Affiliations:** 1 Department of General Medicine, Juntendo University, Tokyo, Japan; 2 Department of Emergency and Critical Care Medicine, Tsukuba Memorial Hospital, 1187-299, Kaname, Tsukuba, Ibaraki, 300-2622 Japan; 3 Department of Health Services Research, Faculty of Medicine, University of Tsukuba, Tsukuba, Japan; 4 Health Services Research and Development Center, University of Tsukuba, Tsukuba, Japan; 5 Division of Trauma and Surgical Critical Care, Osaka General Medical Center, Osaka, Japan; 6 Department of Traumatology and Acute Critical Medicine, Osaka University Graduate School of Medicine, Osaka, Japan; 7 Division of Emergency and Critical Care Medicine, Tohoku University Graduate School of Medicine, Sendai, Japan; 8 Division of Traumatology, Research Institute, National Defense Medical College, Tokorozawa, Japan; 9 Center for General Medicine Education, Keio University School of Medicine, Tokyo, Japan; 10 Trauma and Acute Critical Care Center, Medical Hospital, Tokyo Medical and Dental University, Tokyo, Japan; 11 Division of Disaster and Emergency Medicine, Department of Surgery Related, Kobe University Graduate School of Medicine, Kobe, Japan; 12 Emergency and Critical Care Medicine, Saga University Hospital, Saga, Japan; 13 Department of Emergency and Critical Care Medicine, Keio University School of Medicine, Tokyo, Japan; 14 Department of Acute Medicine, Kawasaki Medical School, Kurashiki, Japan; 15 Advanced Critical Care Center, Aichi Medical University Hospital, Nagakute, Japan; 16 Department of Trauma and Critical Care Medicine, Kyorin University School of Medicine, Tokyo, Japan; 17 Advanced Medical Emergency & Critical Care Center, Yamaguchi University Hospital, Ube, Japan; 18 Department of Emergency and Critical Care Medicine, Chiba University Graduate School of Medicine, Chiba, Japan; 19 Department of Emergency and Critical Care Medicine, St. Luke’s International Hospital, Tokyo, Japan; 20 Division of Acute and Critical Care Medicine, Hokkaido University Graduate School of Medicine, Sapporo, Japan; 21 Department of Acute and Critical Care Medicine, Sapporo Higashi Tokushukai Hospital, Sapporo, Japan

**Keywords:** Frailty, Intensive care units, Infectious disease, Sepsis

## Abstract

**Background:**

Frailty is associated with morbidity and mortality in patients admitted to intensive care units (ICUs). However, the characteristics of frail patients with suspected infection remain unclear. We aimed to investigate the characteristics and outcomes of frail patients with suspected infection in ICUs.

**Methods:**

This is a secondary analysis of a multicenter cohort study, including 22 ICUs in Japan. Adult patients (aged ≥16 years) with newly suspected infection from December 2017 to May 2018 were included. We compared baseline patient characteristics and outcomes among three frailty groups based on the Clinical Frailty Scale (CFS) score: fit (score, 1–3), vulnerable (score, 4), and frail (score, 5–9). We conducted subgroup analysis of patients with sepsis defined as per Sepsis-3 criteria. We also produced Kaplan–Meier survival curves for 90-day survival.

**Results:**

We enrolled 650 patients with suspected infection, including 599 (92.2%) patients with sepsis. Patients with a median CFS score of 3 (interquartile range [IQR] 3–5) were included: 337 (51.8%) were fit, 109 (16.8%) were vulnerable, and 204 (31.4%) were frail. The median patient age was 72 years (IQR 60–81). The Sequential Organ Failure Assessment scores for fit, vulnerable, and frail patients were 7 (IQR 4–10), 8 (IQR 5–11), and 7 (IQR 5–10), respectively (*p* = 0.59). The median body temperatures of fit, vulnerable, and frail patients were 37.5 °C (IQR 36.5 °C–38.5 °C), 37.5 °C (IQR 36.4 °C–38.6 °C), and 37.0 °C (IQR 36.3 °C–38.1 °C), respectively (*p* < 0.01). The median C-reactive protein levels of fit, vulnerable, and frail patients were 13.6 (IQR 4.6–24.5), 12.1 (IQR 3.9–24.9), 10.5 (IQR 3.0–21.0) mg/dL, respectively (p < 0.01). In-hospital mortality did not statistically differ among the patients according to frailty (*p* = 0.19). Kaplan–Meier survival curves showed little difference in the mortality rate during short-term follow-up. However, more vulnerable and frail patients died after 30-day than fit patients; this difference was not statistically significant (*p* = 0.25). Compared with the fit and vulnerable groups, the rate of home discharge was lower in the frail group.

**Conclusion:**

Frail and vulnerable patients with suspected infection tend to have poor disease outcomes. However, they did not show a statistically significant increase in the 90-day mortality risk.

## Background

Frailty is a clinical status and a multidimensional syndrome characterized by the loss of physiologic and cognitive reserves [1, 2]. There are two major approaches to its measurement: the phenotypic frailty model and the frailty index of deficit accumulation [3]. The phenotypic frailty model focuses predominantly on physical symptoms, such as weight loss, exhaustion, weakness, slowness, and reduced physical activity. The frailty index of deficit accumulation focuses on comorbidities, illness, laboratory abnormalities, and functional impairments. Although majority of frailty assessment tools fall into either approach [4, 5], agreement between these tools has been shown to greatly vary [6, 7]. Clinical Frailty Scale (CFS) [1] has been developed as a simple screening tool to assess frailty and has been validated in critical care settings [8, 9].

There is a growing interest in the impact of frailty on patients with critical illness due in part to the increased risk of morbidity and mortality in patients with critical illnesses in intensive care units (ICUs) [8]. Infection in critically ill older adult patients have unique features compared with young patients, wherein the older adults have higher susceptibility to infection [10, 11] and exhibit atypical signs of infection [12, 13]. Moreover, indications for ICU admission of older adult patients remain controversial [14]. However, most previous studies have described the clinical features of frailty in the older adult [15–17] or patients with heterogeneous diseases in ICUs [18–21]. The specific clinical characteristics of frail patients with suspected infection, including sepsis, which is one of the major causes of admission to ICUs, are unknown [22].

Therefore, we aimed to investigate the association between frailty and patient characteristics, clinical features, and outcomes among adult patients with suspected infection in ICUs.

## Methods

### Design and participants

This is a secondary analysis of data from the Japanese Association for Acute Medicine (JAAM) Sepsis Prognostication in Intensive Care Unit and Emergency Room (SPICE) study, a multicenter study of patients with sepsis. The JAAM SPICE study was composed of a SPICE emergency room cohort and a SPICE ICU cohort. We used the SPICE ICU cohort. The SPICE ICU cohort included adult patients (aged ≥16 years) admitted to a participating ICU with a suspected infection. We excluded patients who had missing data on frailty.

### Data collection

Data were collected by the SPICU ICU investigators as part of the routine clinical workup. Data collection methods have been described in a previous study [23]; the investigators entered data into an online standardized template. Patient information included demographic characteristics, admission source, comorbidities, frailty, sites of infection, sepsis-related severity scores including the Sequential Organ Failure Assessment (SOFA) score and the Acute Physiology and Chronic Health Evaluation II score, and laboratory data. In addition, we collected data regarding in-hospital mortality, place after discharge, ventilator-free days (VFDs), ICU-free days (IFDs), and length of hospital stay (LOS).

### Definitions

Suspected infection was defined as the administration of antibiotics and the sampling of any bacterial culture or imaging test undertaken for the purpose of investigating the source of infection. Sepsis and septic shock were defined on the basis of Sepsis-3 criteria [24]. Frailty was evaluated using CFS scores [1]. The CFS score is a 9-point assessment tool used to quantify frailty. Clinicians determined patients’ CFS scores by interviewing them or their surrogates and reviewing their medical records upon admission to the hospital. No training on the use of the CFS score was provided as the score was deemed to be easily understandable by clinicians. Moreover, VFDs were defined as the number of days within the first 28 days after enrollment during which a patient was able to breathe without a ventilator. Patients who died during the study period were assigned a VFD score of 0. IFDs were calculated in a similar manner to the VFDs.

### Analysis

We compared baseline patient characteristics and outcomes, including in-hospital, 30-day, and 90-day mortality, among the three frailty groups based on the CFS score, i.e., fit (score 1–3), vulnerable (score 4), and frail (score 5–9), and evaluated the findings in light of previous reports [15, 25]. The 90-day survival as an outcome was chosen to evaluate differences in survival rates among the groups based on previous studies reporting that frailty might affect long-term survival [18, 21]. Continuous variables were summarized using the median and interquartile range (IQR) and compared using the Kruskal-Wallis test. Categorical variables were summarized using numbers and percentages and compared using the chi-squared test or Fisher exact test, where appropriate. Kaplan–Meier survival curves for 90-day survival were produced and compared using a log-rank test. We conducted a Cox proportional hazards regression analysis to assess the impact of frailty on 90-day survival. Adjusted variables in the analysis included age, sex, the Charlson comorbidity index, and the SOFA score, which were selected on the basis of clinical relevance and previous reports [15, 18]. We tested for interactions between frailty and age, frailty and the Charlson comorbidity index, and age and the Charlson comorbidity index. We also conducted a subgroup analysis of patients diagnosed with sepsis based on Sepsis-3 criteria. A *p*-value of < 0.05 was considered to indicate statistical significance. All statistical analyses were performed with EZR (version 1.38; Saitama Medical Center, Jichi Medical University, Saitama, Japan), a graphical user interface for R (version 3.5.0; The R Foundation for Statistical Computing, Vienna, Austria) [26]. EZR is a modified version of the R commander designed to apply statistical functions that are frequently used in biostatistics.

## Results

**Fig. 1:**
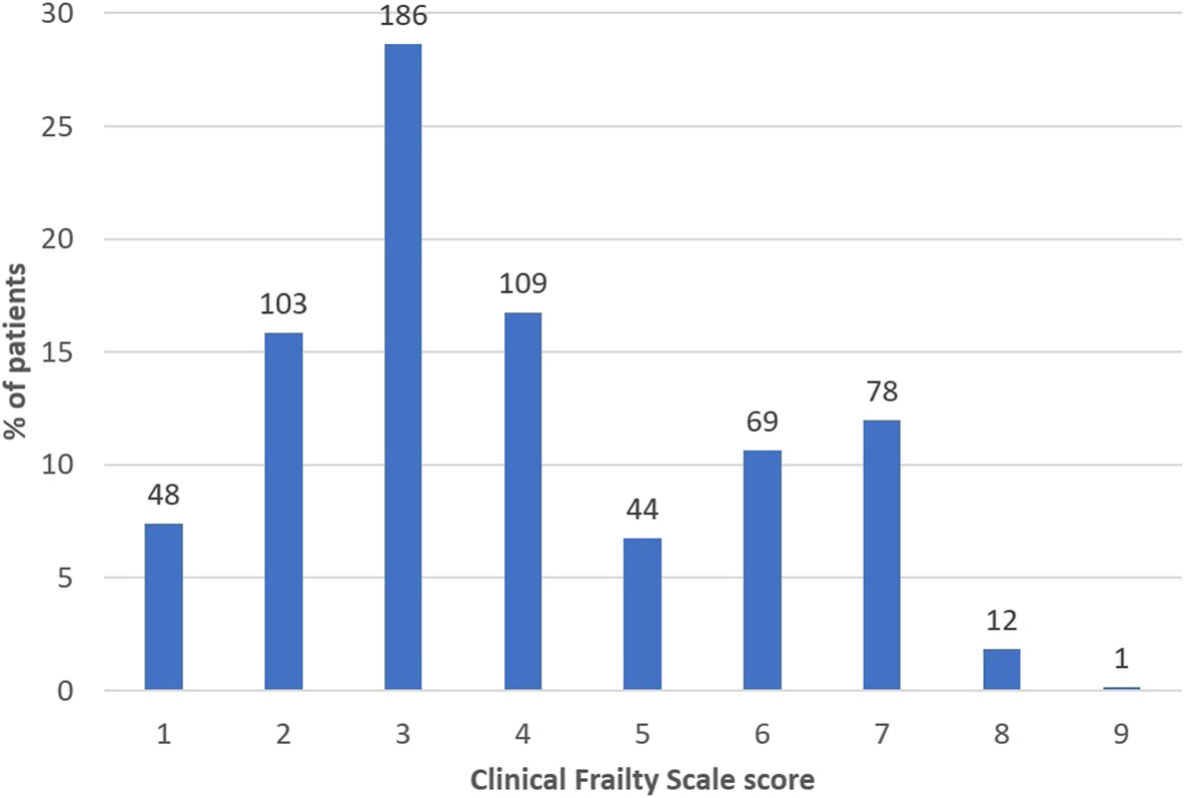
Distribution of Clinical Frailty Scale scores and prevalence of frailty among the enrolled patients. The number at the top of each graph shows the number of patients in each category

We enrolled 650/652 patients with suspected infection from the SPICE ICU database, after excluding 2 patients who had missing data on frailty. The median age of the patients was 72 years (IQR 60–81), and 369 (56.8%) were men. The median CFS score was 3 (IQR 3–5). There were 337 (51.8%) fit patients, 109 (16.8%) vulnerable patients, and 204 (31.4%) frail patients (Table 1 and Fig. 1). The age of patients increased with increasing frailty: fit 67 years (IQR 54–78); vulnerable 73 years (IQR 64–81); and frail 77 years (IQR 69–84), *p* < 0.01. Comorbidities including congestive heart failure, cerebrovascular diseases, dementia, and chronic obstructive pulmonary disease (COPD) were more common in vulnerable and frail patients than in fit patients (p < 0.01). The SOFA scores of fit, vulnerable, and frail patients were 7 (IQR 4–10), 8 (IQR 5–11), and 7 (IQR 5–10), respectively (*p* = 0.59). The patients’ median body temperatures were as follows: fit 37.5 °C (IQR 36.5 °C–38.5 °C); vulnerable 37.5 °C (IQR 36.4 °C–38.6 °C); and frail 37.0 °C (IQR 36.3 °C–38.1 °C), *p* < 0.01. C-reactive protein levels in fit, vulnerable, and frail patients were 13.6 (IQR 4.6–24.5) mg/dL, 12.1 (IQR 3.9–24.9) mg/dL, 10.5 (IQR 3.0–21.0) mg/dL, respectively (*p* = 0.04).

**Table 1:** Characteristics of patients with suspected infection

	Fit (CFS 1–3)	Vulnerable (CFS 4)	Frail (CFS 5–9)	
	*n* = 337 (51.8)	*n* = 109 (16.8)	*n* = 204 (31.4)	*p*-value
Age at admission (years old)	67 (54–78)	73 (64–81)	77 (69–84)	< 0.01
Sex, male	199 (59.1)	68 (62.4)	102 (50.0)	0.05
BMI (kg/m^2^)	22.4 (20.0–25.0)	22.5 (19.6–24.9)	20.8 (17.8–23.6)	< 0.01
Coexisting conditions				
Myocardial infarction	11 (3.3)	7 (6.4)	7 (3.4)	0.33
Congestive heart failure	20 (5.9)	11 (10.1)	28 (13.7)	< 0.01
Peripheral vascular disease	9 (2.7)	7 (6.4)	7 (3.4)	0.17
Cerebrovascular disease	20 (5.9)	9 (8.3)	30 (14.7)	< 0.01
Dementia	12 (3.6)	15 (13.8)	48 (23.5)	< 0.01
COPD	12 (3.6)	13 (11.9)	30 (14.7)	< 0.01
Connective tissue disease	14 (4.2)	13 (11.9)	19 (9.3)	< 0.01
Peptic ulcer disease	13 (3.9)	1 (0.9)	10 (4.9)	0.19
Diabetes mellitus without organ damage	47 (13.9)	22 (20.2)	42 (20.6)	0.09
Diabetes mellitus with organ damage	28 (8.3)	19 (17.4)	14 (6.9)	< 0.01
Chronic kidney disease	19 (5.6)	20 (18.3)	16 (7.8)	< 0.01
Hemiplegia	3 (0.9)	3 (2.8)	25 (12.3)	< 0.01
Malignancy (solid)	30 (8.9)	19 (17.4)	28 (13.7)	0.03
Malignancy (blood)	6 (1.8)	0	1 (0.5)	0.18
Metastatic tumor	6 (1.8)	4 (3.7)	5 (2.5)	0.46
Mild liver disease	8 (2.4)	11 (10.1)	9 (4.4)	< 0.01
Moderate to severe liver disease	13 (3.9)	1 (0.9)	9 (4.4)	0.26
AIDS	0	0	0	
CCI	1 (0–2)	2 (1–4)	2 (1–3)	< 0.01
SOFA score	7 (4–10)	8 (5–11)	7 (5–10)	0.59
APACHE II score	18 (12–25)	22 (17–28)	21 (15–27)	< 0.01
Septic shock	60 (17.8)	23 (21.1)	28 (13.7)	0.22
Mechanical ventilation	132 (39.3)	46 (43.4)	74 (36.5)	0.49
Vital signs				
Glasgow coma scale	13 (8–15)	11 (8–15)	12 (7–14)	< 0.01
Systolic blood pressure (mmHg)	107 (87–128)	105 (80–137)	109 (86–128)	0.97
Heat rate (/min)	105 (88–125)	108 (90–120)	103 (86–118)	0.18
Respiratory rate (/min)	24 (19–29)	22 (18–27)	23 (19–30)	0.42
Body temperature (°C)	37.5 (36.5–38.5)	37.5 (36.4–38.6)	37.0 (36.3–38.1)	0.03
Laboratory data				
White blood cells (/μL)	11,000 (5780–15,580)	10,520 (6700–16,000)	11,780 (7450–17,200)	0.32
Hematocrit (%)	35.4 (29.3–40.8)	33.1 (26.8–39.1)	34.4 (29.4–39.9)	0.07
Platelet (/μL)	16.3 (9.8–24.4)	18.0 (11.2–24.3)	18.1 (12.9–25.5)	0.16
PT-INR	1.2 (1.1–1.4)	1.2 (1.1–1.4)	1.2 (1.1–1.4)	0.83
Lactate (mmol/L)	2.6 (1.4–4.4)	2.7 (1.6–5.7)	2.5 (1.4–4.4)	0.27
Glucose (mg/dL)	142 (112–205)	150 (109–210)	138 (103–194)	0.39
Sodium (mEq/L)	138 (134–141)	138 (135–141)	138 (134–141)	0.94
Potassium (mEq/L)	4.0 (3.6–4.5)	4.0 (3.4–4.7)	4.1 (3.6–4.6)	0.40
Creatinine (mg/dL)	1.5 (0.8–2.6)	1.6 (0.9–2.9)	1.2 (0.7–2.1)	0.02
Total bilirubin (mg/dL)	0.8 (0.5–1.5)	0.8 (0.5–1.5)	0.7 (0.5–1.1)	0.02
C-reactive protein (mg/dL)	13.6 (4.6–24.5)	12.1 (3.9–24.9)	10.5 (3.0–21.0)	0.04
Positive blood cultures	141 (44.2)	49 (47.6)	85 (44.5)	0.84
Site of infection at final diagnosis				
Lung	103 (30.6)	39 (35.8)	81 (39.7)	< 0.01
Abdomen	74 (22.0)	21 (19.3)	35 (17.2)	
Urinary tract	49 (14.5)	13 (11.9)	44 (21.6)	
Soft Tissue	43 (12.8)	18 (16.5)	20 (9.8)	
Others	35 (10.4)	9 (8.3)	7 (3.4)	

Reported counts (proportions) for categorical and median (interquartile range) for continuous variables

Continuous variables were compared using the Kruskal-Wallis test. Categorical variables were compared using the Fisher’s exact test or chi square test, where appropriately

Missing data: BMI = 5; Metastatic tumor = 1; Mechanical ventilation = 2; Systolic blood pressure = 2; Heart rate = 1; Temperature = 1; Hematocrit = 1; PT–INR = 5; Lactate = 15; Glucose = 6; Total bilirubin = 1; C-reactive protein = 2; Positive blood cultures = 37

*CFS* clinical frailty scale, *BMI* body mass index, *COPD* chronic obstructive pulmonary disease, *AIDS* acquired immunodeficiency syndrome, *CCI* Charlson comorbidity index, *SOFA* sequential organ failure assessment, *APACHE* acute physiology and chronic health evaluation, *PT-INR* international normalized ratio of prothrombin time

Table 2 shows the outcomes among fit, vulnerable, and frail patients. There was no statistically significant difference in in-hospital mortality between the three frailty groups: fit 55/335 (16.4%); vulnerable 23/107 (21.5%); and frail 45/203 (22.2%), *p* = 0.19. Likewise, frailty was not associated with 30-day or 90-day mortality. There were no significant differences in IFDs, VFDs, or LOS between the three frailty groups. Frailty was associated with disposition after discharge (discharge to home: fit 125/280 [44.6%]; vulnerable 36/84 [42.9%]; and frail 40/158 [25.3%], *p* < 0.01).

**Table 2:** Outcomes of patients with suspected infection

	Fit (CFS 1–3)	Vulnerable (CFS 4)	Frail (CFS 5–9)	*p*-value
	*n* = 337 (51.8)	*n* = 109 (16.8)	*n* = 204 (31.4)	*p*-value
In-hospital mortality				
Overall	55/335 (16.4)	23/107 (21.5)	45/203 (22.2)	0.19
30-day	40/335 (11.9)	16/107 (15.0)	34/203 (16.7)	0.26
90-day	51/335 (15.2)	22/107 (20.6)	44/203 (21.7)	0.13
Dispositions				
Home	125/280 (44.6)	36/84 (42.9)	40/158 (25.3)	< 0.01
Transfer	155/280 (55.4)	48/84 (57.1)	118/158 (74.7)	
ICU-free days	16 (0–22)	17 (0–22)	15 (0–22)	0.85
Ventilator–free days	21 (0–28)	21 (8–28)	20 (0–28)	0.71
Length of hospital stay	22 (10–49)	23 (14–41)	23 (11–40)	0.86

Reported counts (proportions) for categorical and median (interquartile range) for continuous variables. Continuous variables were compared using the Kruskal-Wallis test. Categorical variables were compared using the Fisher’s exact test or chi square test, where appropriately

Missing data: In–hospital mortality = 5; ICU–free days = 41; Ventilator–free days = 41; Length of hospital stay = 5

*CFS* clinical frailty scale, *ICU* intensive care unit

**Fig. 2:**
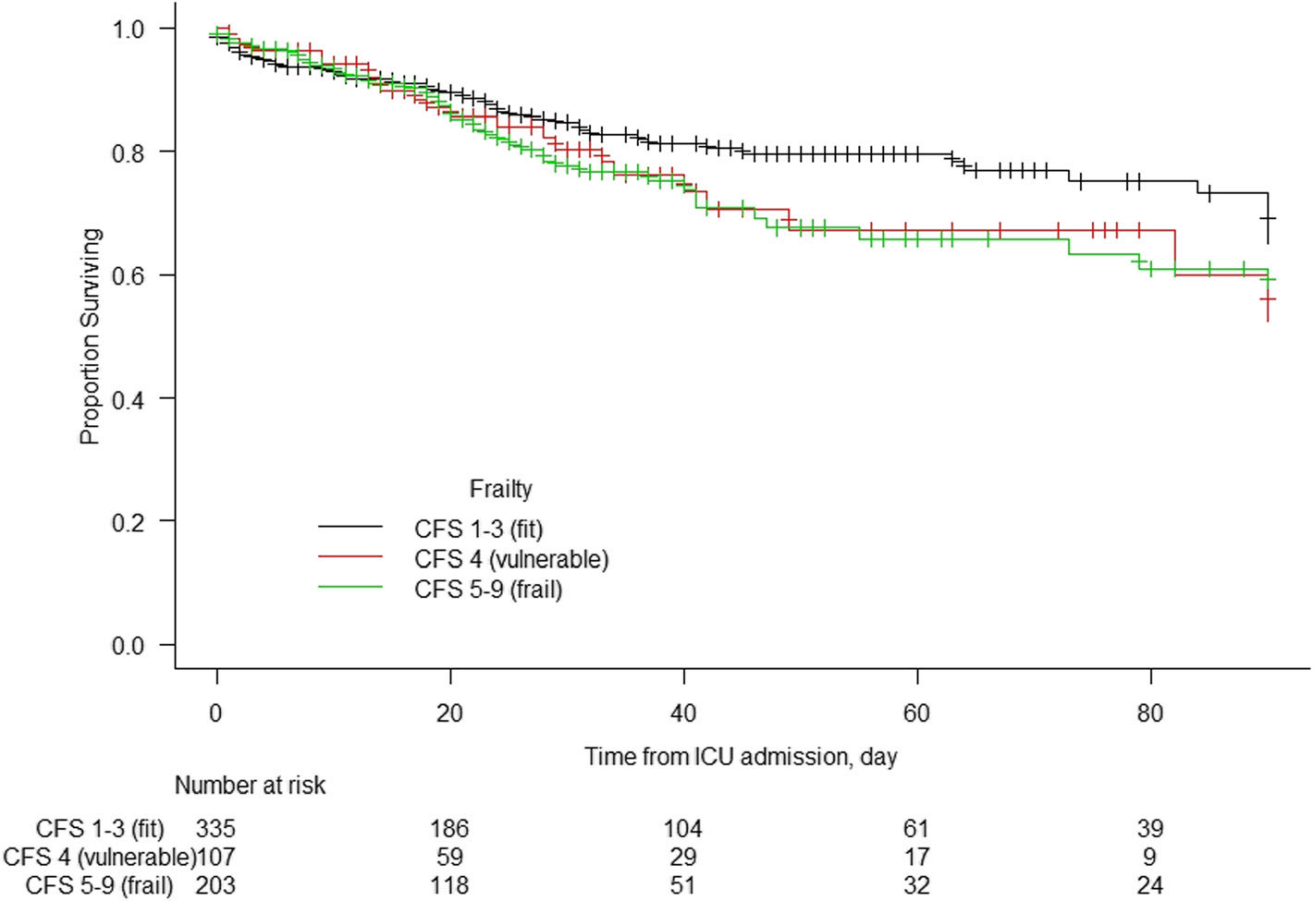
Kaplan–Meier survival curves stratified by the three frailty groups. CFS: Clinical frailty scale. ICU: Intensive care unit

Figure 2 shows the Kaplan–Meier survival curves stratified by the three groups. There was little difference in in-hospital mortality between the groups during 30-day. However, more vulnerable and frail patients died after 30-day phase than did fit patients, although this difference was not statistically significant (*p* = 0.25). Cox proportional hazards regression analysis did not demonstrate an association between in-hospital mortality and frailty (vulnerable vs. fit: adjusted hazard ratio 1.16 [95% confidential interval, 0.70–1.92], *p* = 0.57, frail vs. fit: adjusted hazard ratio 1.13 [95% confidential interval 0.75–1.72], *p* = 0.56), and there were no interactions between frailty and age, frailty and the Charlson comorbidity index, and age and the Charlson comorbidity index (Table 3).

**Table 3:** Univariable and multivariable analysis for mortality associated with frailty in patients with suspected infection

	HR	95% CI		*p*-value
Univariable analysis				
Frailty				
Vulnerable vs fit	1.33	0.82	2.16	0.25
Frail vs fit	1.36	0.92	2.01	0.13
Multivariable analysis				
Age	1.01	1.00	1.03	0.04
Sex, male	1.10	0.76	1.61	0.61
Charlson comorbidity index	1.04	0.95	1.15	0.39
SOFA score	1.18	1.14	1.24	< 0.01
Frailty				
Vulnerable vs fit	1.16	0.70	1.92	0.57
Frail vs fit	1.13	0.75	1.72	0.56

*HR* hazard ratio, *CI* confidence interval*, SOFA* sequential organ failure assessment

Among patients with suspected infection, 599 (92.2%) patients were diagnosed with sepsis. The subgroup analysis of patients with sepsis gave similar results to the primary analysis (Tables 4 and 5). Similarly, there was no association between in-hospital mortality and frailty in patients with sepsis (vulnerable vs. fit: adjusted hazard ratio 1.22 [95% confidential interval, 0.73–2.04], *p* = 0.45, frail vs. fit: adjusted hazard ratio 1.26 [95% confidential interval 0.82–1.93], *p* = 0.29; Table 6).

**Table 4:** Characteristics of patients with sepsis

	Fit (CFS 1–3)	Vulnerable (CFS 4)	Frail (CFS 5–9)	
	303 (50.6)	104 (17.4)	192 (32.1)	*p*-value
Age at admission (years old)	68 (55–78)	73 (64–81)	78 (69–84)	< 0.01
Sex. male	175 (57.8)	66 (63.5)	96 (50.0)	0.06
BMI (kg/m^2^)	22.6 (20.0–25.0)	22.5 (20.0–24.8)	20.8 (17.8–23.3)	< 0.01
Coexisting conditions				
Myocardial infarction	8 (2.6)	7 (6.7)	6 (3.1)	0.14
Congestive heart failure	19 (6.3)	11 (10.6)	26 (13.5)	0.02
Peripheral vascular disease	9 (3.0)	6 (5.8)	6 (3.1)	0.38
Cerebrovascular disease	19 (6.3)	9 (8.7)	29 (15.1)	0.01
Dementia	11 (3.6)	15 (14.4)	47 (24.5)	< 0.01
COPD	11 (3.6)	12 (11.5)	27 (14.1)	< 0.01
Connective tissue disease	12 (4.0)	13 (12.5)	17 (8.9)	0.01
Peptic ulcer disease	13 (4.3)	1 (1.0)	10 (5.2)	0.19
Diabetes mellitus without organ damage	44 (14.5)	21 (20.2)	39 (20.3)	0.18
Diabetes mellitus with organ damage	24 (7.9)	18 (17.3)	12 (6.2)	< 0.01
Chronic kidney disease	17 (5.6)	19 (18.3)	15 (7.8)	< 0.01
Hemiplegia	3 (1.0)	3 (2.9)	24 (12.5)	< 0.01
Malignancy (solid)	26 (8.6)	18 (17.3)	25 (13.0)	0.04
Malignancy (blood)	6 (2.0)	0	1 (0.5)	0.24
Metastatic tumor	6 (2.0)	4 (3.8)	5 (2.6)	0.57
Mild liver disease	8 (2.6)	11 (10.6)	7 (3.6)	< 0.01
Moderate to severe liver disease	12 (4.0)	1 (1.0)	9 (4.7)	0.25
AIDS	0	0	0	
CCI	1 (0–2)	2 (1–4)	2 (1–3)	< 0.01
SOFA score	8 (5–11)	8 (5–11)	7 (5–10)	0.75
APACHE II score	19 (14–26)	24 (18–28)	21 (15–28)	< 0.01
Septic shock	59 (19.5)	23 (22.1)	27 (14.1)	0.17
Mechanical ventilation	120 (39.6)	46 (45.1)	69 (35.9)	0.31
Vital signs				
Glasgow coma scale	13 (7–15)	11 (8–14)	11 (7–14)	< 0.01
Systolic blood pressure (mmHg)	105 (85–127)	100 (79–132)	109 (86–128)	0.88
Heat rate (/min)	106 (90–126)	108 (90–121)	104 (86–119)	0.19
Respiratory rate (/min)	24 (19–30)	22 (18–27)	24 (19–30)	0.17
Body temperature (°C)	37.5 (36.6–38.5)	37.3 (36.4–38.5)	37.1 (36.3–38.2)	0.04
Laboratory data				
White blood cells (/μL)	11,000 (5650–15,895)	10,555 (6625–15,925)	11,660 (7568–17,250)	0.35
Hematocrit (%)	35.5 (29.5–40.8)	33.1 (26.8–39.2)	34.3 (29.3–39.9)	0.04
Platelet (/μL)	15.9 (9.8–23.7)	16.8 (11.0–24.2)	18.0 (12.8–25.5)	0.10
PT-INR	1.2 (1.1–1.4)	1.2 (1.1–1.4)	1.2 (1.1–1.4)	0.92
Lactate (mmol/L)	2.6 (1.5–4.8)	2.7 (1.7–5.9)	2.7 (1.6–4.4)	0.43
Glucose (mg/dL)	139 (110–205)	144 (108–204)	136 (102–194)	0.44
Sodium (mEq/L)	137 (134–141)	137 (135–141)	138 (134–142)	0.59
Potassium (mEq/L)	4.0 (3.6–4.6)	4.0 (3.4–4.7)	4.1 (3.6–4.6)	0.53
Creatinine (mg/dL)	1.5 (0.9–2.8)	1.6 (0.9–3.0)	1.3 (0.7–2.1)	0.01
Total bilirubin (mg/dL)	0.9 (0.6–1.5)	0.9 (0.5–1.5)	0.7 (0.50–1.1)	0.01
C-reactive protein (mg/dL)	14.4 (5.4–24.7)	12.2 (4.0–25.3)	11.1 (3.0–21.1)	0.03
Positive blood cultures	135 (46.6)	47 (47.5)	82 (45.6)	0.95
Site of infection at final diagnosis				
Lung	90 (29.7)	38 (36.5)	79 (41.1)	< 0.01
Abdomen	67 (22.1)	20 (19.2)	32 (16.7)	
Urinary tract	47 (15.5)	13 (12.5)	41 (21.4)	
Soft Tissue	36 (11.9)	16 (15.4)	18 (9.4)	
Others	33 (10.9)	8 (7.7)	7 (3.6)	

Reported counts (proportions) for categorical and median (interquartile range) for continuous variables

Continuous variables were compared using the Kruskal-Wallis test. Categorical variables were compared using the Fisher’s exact test or chi square test, where appropriately

Missing data: BMI = 5; Metastatic tumor = 1; Systolic blood pressure = 2; Heart rate = 1; Temperature = 1; Hematocrit = 1; PT-INR = 2; Lactate = 9; Glucose = 4; Total bilirubin = 1; C–reactive protein =1; Positive blood cultures = 30

*CFS* clinical frailty scale, *BMI* body mass index, *COPD* chronic obstructive pulmonary disease, *AIDS* acquired immunodeficiency syndrome, *CCI* Charlson comorbidity index, *SOFA* sequential organ failure assessment, *APACHE* acute physiology and chronic health evaluation, *PT–INR* international normalized ratio of prothrombin time

**Table 5:** Outcomes of patients with sepsis

	Fit (CFS 1–3)	Vulnerable (CFS 4)	Frail (CFS 5–9)	
	303 (50.6)	104 (17.4)	192 (32.1)	*p*-value
In-hospital mortality				
Overall	51/302 (16.9)	23/102 (22.5)	44/191 (23.0)	0.18
30-day	38/302 (12.6)	16/102 (15.7)	34/191 (17.8)	0.26
90-day	47/302 (15.6)	22/102 (21.6)	43/191 (22.5)	0.11
Dispositions				
Home	110/251 (43.8)	34/79 (43.0)	36/147 (24.5)	< 0.01
Transfer	141/251 (56.2)	45/79 (57.0)	111/147 (75.5)	
ICU–free days	15 (0–21)	16 (0–21)	14 (0–22)	0.83
Ventilator–free days	21 (0–28)	21 (6–28)	20 (0–28)	0.87
Length of hospital stay	23 (10–49)	23 (14–40)	23 (11–40)	0.98

Reported counts (proportions) for categorical and median (interquartile range) for continuous variables

Continuous variables were compared using the Kruskal-Wallis test. Categorical variables were compared using the Fisher’s exact test or chi square test, where appropriately

Missing data: In-hospital mortality = 4; ICU–free days = 40; Ventilator–free days = 40; Length of hospital stay = 4

*CFS* clinical frailty scale, *ICU* intensive care unit

**Table 6:** Univariable and multivariable analysis for mortality associated with frailty in patients with sepsis

	HR	95% CI		*p*-value
Univariable analysis				
Frailty				
Vulnerable vs fit	1.41	0.86	2.30	0.18
Frail vs fit	1.40	0.94	2.10	0.10
Multivariable analysis				
Age	1.01	1.00	1.03	0.04
Sex. male	1.15	0.78	1.68	0.49
Charlson comorbidity Index.	1.05	0.95	1.16	0.32
SOFA score	1.20	1.15	1.26	< 0.01
Frailty				
Vulnerable vs fit	1.22	0.73	2.04	0.45
Frail vs fit	1.26	0.82	1.93	0.29

*HR* hazard ratio, *CI* confidence interval*, SOFA* sequential organ failure assessment

## Discussion

We investigated the association between frailty and clinical characteristics and outcomes among patients with suspected infection in ICUs. One strength of the present study is the focus on older adult patients with suspected infection in Japan, one of the leading aging countries. The results of our study provide insights for use by societies with impending aging populations. Approximately one-third of the patients were classified as frail according to the CFS score. Frail patients were more likely to be older and had more comorbidities; they were also less likely to be discharged home and had lower temperature and C-reactive protein levels. Vulnerable and frail patients appeared to have poor 30-day outcomes compared with fit patients, although they did not appear to have a statistically significant increased 90-day mortality risk.

As many previous studies have reported, our study showed an increase in frailty with aging. The proportion of older adult patients in our study was higher than that in previous studies regarding frailty; the median age of patients in our study was 72 years; in other studies, the median age was 62 [18] and 64 years [19]. The presence of higher proportion of older adult patients in our study could be because Japan has one of the world’s oldest populations [27]. Another explanation may be that our cohort included a large proportion of patients with sepsis [28]. Regarding the prevalence of frailty, our finding was comparable to previous studies [18, 21, 29] that included ICU populations. The prevalence of frailty varies widely across studies based on patient age [19, 20, 25]. Studies that include a large number of very old patients have a higher prevalence of frailty [15]. The diversity in study population, setting, and study design may have contributed to the different characteristics of frail populations.

We confirmed that frail and vulnerable patients had more comorbidities compared with fit patients. Comorbidities included congestive heart failure, cerebrovascular diseases, and COPD as well as those described in previous studies [30, 31]. Our results were very similar to previous reports that included heterogeneous diseases, although we selected patients with suspected infection only. There exists a controversy regarding the relationship between individual comorbidities and frailty [32]. The combination of individual comorbidities and frailty may not be related to the primary disease, although it is natural that more comorbidities lead to greater frailty.

Our findings with regard to body temperature and C-reactive protein levels suggest that frailty may be associated with a poor acute inflammatory response. The older adults often have an absent or diminished febrile response to infections [13]. Some studies have reported that frailty was associated with chronic changes in the immune response, including the imbalance of decline in immune function and increased inflammation [33, 34]. Other studies have reported that aging was related to changes in the acute immune response [13, 35], due to dysfunction of immune cells or decreased cytokines working as part of innate and adaptive immunity [35]. Both frailty and aging may be involved in weakening of the acute inflammatory response. However, a blunted response was not observed in white blood cell or platelet count in frail patients. Differences in pathophysiological mechanisms and kinetics might have contributed to the differences observed in white blood cell count and C-reactive protein level changes [36]. Further studies are needed to clarify the relationship between frailty, aging, and poor inflammatory responses.

Regarding mortality, we found that more vulnerable and frail patients died after 30-day, although this difference was not statistically significant. This tendency was consistent with some previous reports [1, 18]. In 30-day, disease severity may have had a greater impact on mortality than frailty. We did not observe the patients’ status after discharge, and frail patients who transferred to other institutions might have subsequently died. However, the short-term outcomes in our study were not in agreement with those reported by Fernando et al. [29] Several differences between the studies might explain this discordance. First, the overall mortality was higher (37.0%) and the median hospital stay was shorter (13 days in frail patients and 9 days in non-frail patients) in the study by Fernando et al., indicating that our study included patients with less severe clinical conditions. In addition, differences in the follow-up period might have affected the results. We did not follow patient outcomes after discharge, even those who were discharged to another facility in the early phase. Last observation carry-forward might have contributed to better outcomes. Moreover, the Japanese universal health care system might have contributed to lower mortality in frail patients [37]. Death with dignity for benign diseases has not yet been well understood in Japan. Frail patients tend to be treated at a lower cost if they are admitted to tertiary centers rather than chronic care hospitals, regardless of their quality of life after treatment. Alternatively, the relationship between the severity of frailty and mortality may not have been linear among patients with sepsis. Mortality from septic shock is very high [22]. Vulnerable and frail patients may have already been at risk of death. Further studies are needed to assess the association between the severity of frailty and mortality in patients with sepsis.

### Limitations

This study had some limitations. First, fewer patients had CFS scores of 5 in our study compared with those in previous studies [15, 18, 21]. In addition, analysis for data reliability was not performed. Moreover, mild dementia is generally observed in patients with a CFS score of 5 according to the original study. However, 3.6% of fit patients had dementia according to the Charlson comorbidity index, and the possibility of misclassification remains. However, in the study that introduced CFS [1], 3.7% of patients with a CFS score of 1 had dementia, similar to that observed in our study. The CFS score is not widely used to assess frailty in Japan. Education in the use of the CFS score may have been necessary although CFS has been found to be a reliable tool even if the assessor is different [38]. Second, we did not have information about treatments that may have been related to the patients’ outcomes in this database. However, most patients should have received appropriate treatments according to guidelines such as the Surviving Sepsis Campaign Guideline, which is used in national certified ICUs [39]. Third, we did not have information on delirium. Because of the high association between frailty and delirium, this unreported factor might have introduced bias to a higher degree in this population.

## Conclusions

Among patients admitted to ICUs with suspected infection, frail patients were more likely to be older and have more comorbidities; frail patients were also less likely to be discharged home and had lower temperature and C-reactive protein levels. However, frail patients did not have a statistically significant increased 90-day mortality risk.

## Data Availability

The datasets analyzed during the current study is available with the corresponding author on reasonable request.

## Abbreviations

CFS: Clinical frailty scale
COPD: Chronic obstructive pulmonary disease
ICUs: Intensive care units
IFDs: ICU-free days
IQR: Interquartile range
JAAM: Japanese Association for Acute Medicine
LOS: Length of hospital stay
SOFA: Sequential organ failure assessment
SPICE: Sepsis Prognostication in Intensive Care Unit and Emergency Room
VFDs: Ventilator-free days
